# Siponimod Induces Peripheral T Cell Redistribution Without Affecting Glial Cells or Behavior in Naïve Mice

**DOI:** 10.3390/brainsci16101060

**Published:** 2026-09-30

**Authors:** Julie Damgaard Rosgaard Jakobsen, Estrid Thougaard, Lejla Vahl Becirovic, Victoria Phuong, Amalie Forsberg Jensen, Tim Wellinghof, Pernille Vinther Nielsen, Stefano Raffaele, Marta Fumagalli, Åsa Fex Svenningsen, Bente Finsen, Agnieszka Wlodarczyk, Flemming Nielsen, Kate Lykke Lambertsen, Helle Hvilsted Nielsen

**Affiliations:** 1 Department of Neurology, Odense University Hospital, 5000 Odense, Denmark; jdrchristensen@health.sdu.dk (J.D.R.J.);; 2 Department of Neurobiology Research, Institute of Molecular Medicine, University of Southern Denmark, 5000 Odense, Denmarkaasvenningsen@health.sdu.dk (Å.F.S.); bfinsen@health.sdu.dk (B.F.);; 3 BRIDGE—Brain Research Inter-Disciplinary Guided Excellence, Department of Clinical Research, University of Southern Denmark, 5000 Odense, Denmark; 4 Department of Pharmaceutical Sciences, Università degli Studi di Milano, 20122 Milano, Italymarta.fumagalli@unimi.it (M.F.); 5 Clinical Pharmacology, Pharmacy and Environmental Medicine, Department of Public Health, University of Southern Denmark, 5000 Odense, Denmark

**Keywords:** siponimod, sphingosine-1-phosphate receptor, lymphocyte trafficking, T cell lymphopenia, neuroimmunology

## Abstract

**Highlights:**

**What are the main findings?**

- Siponimod induced pronounced peripheral T cell lymphopenia and altered lymphocyte distribution across lymphoid organs, with increased splenic T cell populations.
- Despite readily penetrating the blood–brain barrier, siponimod did not measurably affect glial cell densities, microglial morphology, or behavior in naïve mice.

**What are the implications of the main findings?**

- Under physiological conditions, siponimod appears to act predominantly through peripheral immunomodulation rather than through direct effects on CNS resident glial cells.
- The neuroprotective and remyelinating effects of siponimod reported in disease settings may depend on inflammatory processes or immune–glia interactions.

**Abstract:**

**Background**: Siponimod is a selective modulator of sphingosine-1-phosphate receptors 1 and 5, approved for the treatment of secondary progressive multiple sclerosis. Apart from its immunomodulatory effects, it has been proposed to exert pro-regenerative and pro-myelinating effects. However, it is unclear whether the reported neuroprotective and pro-regenerative effects of siponimod reflect direct engagement on glial cells or arise secondary to reduced neuroinflammation. **Methods**: We investigated the systemic and central effects of siponimod in naïve mice treated orally with 3 mg/kg for 19 consecutive days. Peripheral immune cell populations were analyzed by flow cytometry, drug exposure was quantified by liquid chromatography and high-resolution mass spectrometry, glial cell populations were assessed by immunohistochemistry, and functional outcomes were evaluated using behavioral tests. **Results**: Siponimod treatment induced pronounced T cell lymphopenia, characterized by reduced CD8^+^ T cells and increased proportions of double-negative T cells and CD4^+^CD25^+^ T cells. Lymphocyte distribution was compartment-specific, with increased splenic T cell and reduced thymic T cell counts, while lymph nodes were largely unaffected. Siponimod penetrated the CNS, with hippocampal concentrations exceeding plasma levels. Despite robust brain exposure, siponimod did not affect oligodendrocyte lineage cell density, proliferation, microglial cell density, or morphology, nor did it affect behavior in mice. **Conclusions**: Taken together, these findings demonstrate that under physiological conditions, the actions of siponimod lie primarily in its immunomodulatory effects, while direct effects on glial cells and behavior are absent. Furthermore, this study paves the way for studies of siponimod’s effect on glial cells and neurons in models with minimal or no blood–brain barrier damage.

## 1. Introduction

Siponimod is a selective sphingosine-1-phosphate receptor (S1PR) 1 and 5 modulator and has been shown to reduce disability progression in patients with secondary progressive multiple sclerosis (SPMS) [1]. In a randomized clinical trial, siponimod significantly reduced neurological and disability progression, as well as brain atrophy and cognitive function in SPMS patients [1]. Disease progression in the progressive forms of multiple sclerosis (MS) is largely driven by smoldering inflammation behind an intact blood–brain barrier (BBB), leading to diffuse chronic demyelination, progressive axonal loss, and impaired remyelination capacity [2,3,4]. S1PRs regulate a wide range of biological processes, including immune cell trafficking. S1PR1 is expressed on lymphocytes, and its activation induces receptor internalization and degradation, which prevents lymphocyte egress from lymphoid organs [5,6]. This results in reduced numbers of circulating lymphocytes and consequently diminished central nervous system (CNS) inflammation, a mechanism also observed in patients treated with siponimod [7,8]. The drug not only reduces the number of circulating lymphocytes but also alters their composition. While both CD4^+^ and CD8^+^ T cell proportions, as well as the proportion of B cells, were decreased in blood samples from siponimod-treated SPMS patients, the proportion of regulatory T cells was found to be increased, thereby possibly promoting a more anti-inflammatory environment [7,9]. Furthermore, S1PRs are expressed on cells within the CNS. S1PR1 is expressed on oligodendrocyte lineage cells, microglia, astrocytes, and neurons, while S1PR5 expression is largely restricted to oligodendrocyte lineage cells. Siponimod has therefore been proposed to exert direct effects on glial cells within the brain [10].

Previous studies have demonstrated that siponimod exerts protective and pro-regenerative effects on microglia and oligodendrocyte lineage cells in models of demyelination [11,12,13,14]. Effects include preservation of myelin, reduced neurodegeneration, and increases in oligodendrocyte populations [11,12,13,15,16,17]. Siponimod has additionally been associated with reduced microglial numbers and a shift toward a more regenerative microglial phenotype [12,13,17]. Together, these findings suggest that siponimod beneficially modulates glial cell responses under pathological conditions, although it remains unclear whether these effects arise from direct actions on CNS-resident cells or occur secondary to reduced inflammation.

Several analyses presented in this manuscript were done in the hippocampal area. The hippocampus was chosen as the region of interest because it is affected by MS pathology and is closely linked to cognitive functions that are frequently impaired in MS [18,19,20]. Furthermore, hippocampal lesions are common in MS patients [21,22]. Thus, although MS pathology is widespread throughout the CNS, the hippocampus represents a clinically relevant region in which to investigate potential effects of siponimod on microglia and oligodendrocyte lineage cells, especially in relation to behavioral assessments.

Despite the well-established clinical efficacy of siponimod in SPMS, its direct effects on CNS-resident cells remain poorly defined. Most preclinical studies have been performed in inflammatory or demyelinating models, where peripheral immune modulation confounds the assessment of direct CNS actions. Consequently, it is unclear whether the reported neuroprotective and pro-regenerative effects of siponimod reflect direct engagement on glial cells or arise secondary to reduced neuroinflammation. Moreover, the baseline effects of siponimod on immune cell distribution, microglial homeostasis, oligodendrocyte lineage cell functions, and behavior under physiological conditions have not yet been systematically characterized. The objective of the present study was therefore to determine the systemic and CNS-specific effects of siponimod in naïve mice in the absence of disease-induced pathology, with particular focus on peripheral immune cell composition, microglia, and oligodendrocyte lineage cells. We hypothesized that siponimod would alter peripheral immune cell distribution and exert measurable effects on microglia and oligodendrocyte lineage cell homeostasis in the absence of ongoing neuroinflammation or demyelination.

## 2. Materials and Methods

### 2.1. Animals

Experiments were performed using female C57BL/6JRj mice from Janvier Labs (Le Genest-Saint-Isle, France), aged 8–9 weeks. Young adult female mice were selected to reflect the female predominance observed in MS [23]. Mice were kept on a 12 h dark/light cycle with controlled temperature and free access to food and water. All animal experiments and maintenance were approved by The Danish Animal Experiments Inspectorate under the Ministry of Food and Agriculture (J. no. 2021-15-0201-00977, acceptance date: 25 October 2021).

### 2.2. Study Design and Pharmacological Treatment

**Figure 1:**
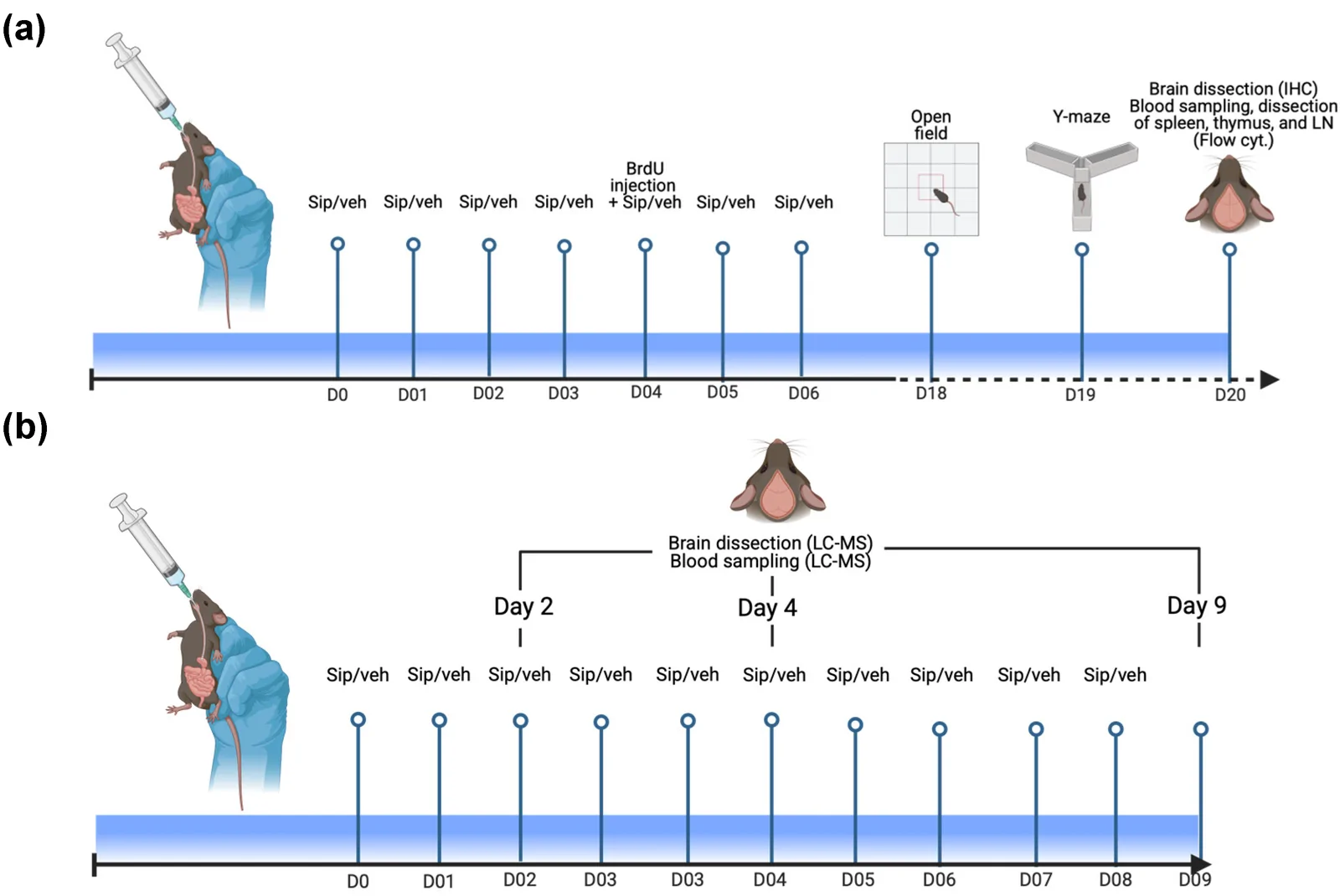
Overview of experiments. (**a**) Mice used for peripheral immune cell analysis, immunohistochemistry, and behavioral analysis were treated with siponimod (3 mg/kg) or vehicle for 19 consecutive days via oral gavage. On day 4, all animals received a single bolus of BrdU (50 mg/kg). Open field was performed on day 18, and Y-maze on day 19. On day 20, all animals were sacrificed. (**b**) Mice used for concentration measurements were treated every day with vehicle or siponimod (3 mg/kg) for 2, 4 or 9 days before sacrifice. Created in BioRender. Lambertsen, K.L. (2026) https://BioRender.com/do2hdfy. Sip, siponimod; D, day; IHC, immunohistochemistry; flow cyt., flow cytometry; LC-MS, liquid chromatography–mass spectroscopy; BrdU, bromodeoxyuridine.

**Figure A1:**
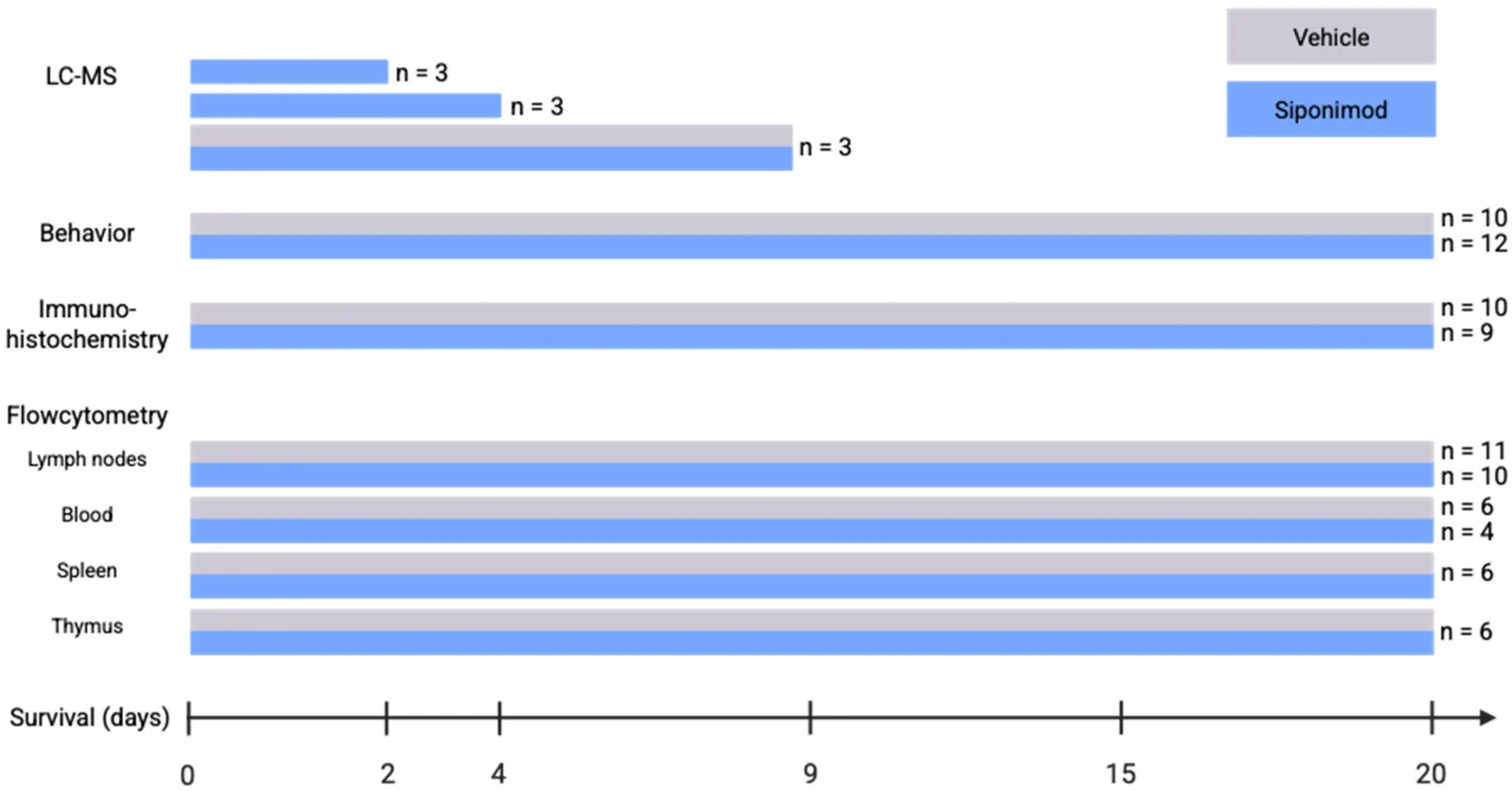
n-values of experiments. The figure presents an overview of n-values and survival in each experiment performed.

A total of 37 mice (Figure A1) were treated once daily by oral gavage with either siponimod fumarate (3 mg/kg; DC Chemicals, DC32397, Shanghai, China) in 1% aqueous sodium carboxymethyl cellulose (Na-CMC; Merck, C4888, Darmstadt, Germany) (*n* = 15) or vehicle (1% Na-CMC, *n* = 22) (Figure 1). This dosing regimen has previously been shown to be effective in other rodent studies [17,24,25]. Based on conventional body surface area scaling, this corresponds to an approximate human-equivalent dose of 0.24 mg/kg [26]. However, this conversion does not account for interspecies differences in pharmacokinetics and should therefore not be interpreted as equivalent to the clinical maintenance dose used in patients with MS. Proliferating cells were labeled with a single intraperitoneal (i.p.) injection of the proliferation marker bromodeoxyuridine (BrdU; 50 mg/kg; Merck, B5002-1G) in isotonic saline on day 4 after treatment initiation. Mice allocated for immunohistochemistry, flow cytometry, and behavioral testing were treated for 19 consecutive days (Figure 1a). For pharmacokinetic analyses, separate cohorts of mice received vehicle for 9 days or siponimod for 2, 4, or 9 days (*n* = 3 mice/group) (Figure 1b). Sample sizes were determined based on the planned experimental design, previous experience with the respective methods, and the number of animals required to obtain sufficient biological replicates while minimizing animal use. For the main experimental analyses, we aimed for approximately eight animals per treatment group. Actual sample sizes varied between analyses because different subsets of animals were allocated to specific assays and because some samples were unavailable or excluded due to technical or tissue-quality issues. Separate pharmacokinetic cohorts comprised three animals per group, while larger group sizes were used for behavioral and histological analyses where feasible. Exact sample sizes for each analysis are reported in the corresponding figures and figure legends. Animals were randomly allocated to the vehicle- and siponimod-treated groups. No computer-generated randomization sequence was used. To minimize potential order effects, animals were handled and treated in a non-systematic order each day. No additional formal measures were used to control for potential confounders related to animal or cage location. All analyses were conducted by an investigator blinded to experimental groups. Two siponimod-treated mice were euthanized during oral gavage and were excluded from this study, while one brain from a vehicle-treated mouse was unavailable for histology, although the animal remained included in blood flow cytometry. An additional three mice were excluded from immunohistochemical analyses due to technical issues. Furthermore, twelve animals were excluded from NG2 analyses and three from morphological analyses because of insufficient tissue quality, preventing reliable quantification.

### 2.3. Behavioral Testing

Locomotor activity and short-term spatial memory were tested using the open field and Y-maze tests on days 18 and 19, respectively, in vehicle- (*n* = 12) and siponimod-treated (*n* = 10) mice.

#### 2.3.1. Open Field Test

The open field test was conducted on day 18 prior to oral treatment. The open field maze consisted of a square arena (45 × 45 cm) enclosed by 45 cm-high walls. At the start of the test, a single mouse was placed in the center of the maze and allowed to explore freely for 10 min [27]. Animal movement was recorded and analyzed using the SMART 3.0 tracking system (Panlab, Harvard Apparatus, Barcelona, Spain). For analysis, the maze was subdivided into three zones: a peripheral wall zone, an intermediate (interperi) zone, and a central zone corresponding to the innermost quadrant. The zones were used to quantify total distance traveled (cm) and the center-to-perimeter ratio (time spent in peripheral zones (s)/time spent in the center (s)), an index of anxiety-like behavior.

#### 2.3.2. Y-Maze

The Y-maze test was conducted using a transparent Y-shaped maze consisting of three identical arms positioned 120° apart. On day 19 of the experiment, each mouse was placed at the center of the maze and allowed to explore freely for 10 min. Arm entries and their sequence were recorded manually by an experimenter blinded to treatment groups. An arm entry was defined as the mouse placing all four limbs within an arm. The first two arm entries were excluded from analysis to account for the animal’s initial placement and orientation. A spontaneous alternation was defined as consecutive entries into all three arms without repetition (e.g., ABC, BCA, or CAB). Outcomes were reported as total number of entries and percentage of spontaneous alternation behavior (%SAB), calculated as: %SAB = (number of spontaneous alternations/(total arm entries − 2)) × 100 [28].

### 2.4. Tissue Collection

At the end of the experiment, mice were euthanized by a single i.p. injection with pentobarbital (200 mg/mL, VetViva, 182831, Wels, Austria) combined with lidocaine (10 mg/mL, AstraZeneca, 520455, Copenhagen, Denmark). Prior to perfusion, blood samples for flow cytometry were collected using heparin-coated syringes and transferred to EDTA-coated tubes. Mice were then perfused with 0.5 M tris-buffered saline (TBS) and inguinal lymph nodes, spleen, and thymus were collected for flow cytometric analysis. Following perfusion with 4% paraformaldehyde (PFA) (Santa Cruz Biotechnology, Sc-281692, Dallas, TX, USA), brains were dissected and post-fixed in 4% PFA for 1 h, after which they were transferred to 1% PFA. On the following day, brains were transferred to and stored in 0.1% PFA until sectioning into 50 µm thick horizontal serial sections using a vibratome (Leica, VT1000S, Wetzlar, Germany). Brain sections were stored in De Olmos cryoprotectant solution (700 mL dH_2_O, 9.5 g Na_2_HPO_4_.2H_2_O (Merck, 10028.24-7), 2.12 g NaH_2_PO_4_.H_2_O (Merck, 10049-21-5), 300 mL ethylene glycol (Merck, 107-21-1), 300 g sucrose (Merck, 57-50-1), and 10 g PVP40 (Sigma-Aldrich, 9003-39-8, Darmstadt, Germany)) at −12 °C. For siponimod concentration measurements, blood was collected as described above and centrifuged at 10,000× *g* for 10 min to obtain plasma. Mice were subsequently perfused with phosphate-buffered saline (PBS), after which hippocampi were dissected, snap-frozen, and stored at −80 °C until further processing.

### 2.5. Flow Cytometry

For flow cytometric analysis of immune cell populations, lymph nodes (vehicle, *n* = 8; siponimod, *n* = 9), spleen (vehicle, *n* = 6; siponimod, *n* = 6), and thymus (vehicle, *n* = 6; siponimod, *n* = 6) were mechanically dissociated through a 70 µm cell strainer (Falcon, Corning, NY, 352350, USA) into RPMI-1640 medium (ThermoFischer, A1049101, Waltham, MA, USA). Blood samples (vehicle, *n* = 6; siponimod, *n* = 4) were subjected to erythrocyte lysis using distilled water, washed in PBS, and resuspended in Dulbecco’s PBS (D-PBS; Thermo Fischer, 14190144). The following steps apply to all tissue types: isolated cells were stained with a fixable viability dye eFlourTM506 (eBioscience, 65-0866-14, San Diego, CA, USA, 1:1000 in D-PBS) for 30 min followed by Fc receptor blocking in using Syrian hamster gamma globulin (50 µg; Jackson ImmunoResearch, 007-000-002, West Grove, PA, USA) and rat anti-mouse CD16/CD32 Fc Block (BD Biosciences, 553141, Franklin Lakes, NJ, USA) in Fluorescence-activated cell sorting (FACS) buffer for 30 min. FACS buffer consisted of Hank’s balanced salt solution (HBSS: 0.14 M NaCl, 5.4 mM KCl, 0.4 mM MgSO_4_•7H_2_O, 0.4 mM Na_2_HPO_4_ (anhydrous), 1.3 mM CaCl_2_2H_2_O, 4.2 mM NaHCO_3_, 0.4 mM KH_2_PO_4_, 0.5 mM MgCl_2_6H_2_O, and 5 mM glucose) supplemented with 2% fetal bovine serum (FBS; Sigma-Aldrich, F9665) and 0.1% sodium azide. Cells were subsequently stained with fluorophore-conjugated antibodies against surface antigens for 30 min (Table A1), fixed in 1% PFA for 30 min, and washed before resuspension in FACS buffer containing 5000 Trucount^TM^ beads (BD Biosciences, 663028) per sample. Data acquisition was performed on a FACSymphony A1 flow cytometer (BD Biosciences), and data were analyzed using FlowJo software v.10 (FlowJo LLC, Franklin Lakes, NJ, USA). The gating strategy is shown in Section 3.1.

### 2.6. Analysis of Siponimod Concentrations in Brain Tissue and Plasma

Siponimod concentrations in hippocampal tissue (vehicle, *n* = 3; siponimod, *n* = 9) and plasma samples (vehicle, *n* = 3; siponimod, *n* = 8) were determined using liquid chromatography and high-resolution mass spectrometry (LC-HRMS). The LC-HRMS system consisted of a Vanquish Ultra high-pressure liquid chromatography (UHPLC) coupled to a Q-Exactive mass spectrometer (Thermo Scientific, Waltham, MA, USA). Siponimod was detected in positive ionization mode at *m*/*z* 517.2675, monitored at a resolution of 70.000, with automated gain control, a full target scan at 1 × 10^6^, and maximum IT (Orbitrap filling time) at 200 ms. Chromatographic separation was performed using an ACE Excel 3 C18-AR 50 × 2.1 mm column (Avantor ACE, Aberdeen, Scotland), operated at a temperature of 40 °C, and eluted with a gradient of 0.1% formic acid in Milli-Q water (eluent A) and 0.1% formic acid in acetonitrile (eluent B) delivered at a flow rate of 0.4 mL/min. The gradient elution program was as follows: 0–0.7 min, 50% B; 0.7–5.0 min, linear increase from 50% to 95% B; 5.0–6.0 min, 95% B; and 6.0–7.0 min, re-equilibration to 50% B. A sample volume of 5 µL was injected into the column. Quantification was performed using an external calibration curve prepared in blank plasma over a concentration range from 1–1000 ng/mL. For plasma samples, the calibration curve was described by Y = −1.11732 × 10^6^ + 502836X (R^2^ = 0.9979), whereas for hippocampal samples the regression equation was Y = 47843.6 + 500027X (R^2^ = 0.9985).

The calibration curve was linear and weighted 1/x, with a coefficient of determination (r^2^) of 0.998 for plasma and 0.999 for hippocampal lysates.

#### Sample Preparation

Plasma samples and calibrators (100 µL) were transferred to 1.5 mL Eppendorf tubes, followed by the addition of 25 µL of 40% formic acid. Samples were vortexed for 15 s, after which 600 µL of a mixture consisting of acetonitrile, methanol, and acetic acid (90:10:0.1, *v*/*v*/*v*) was added. Following addition of the precipitation solvent, samples were continuously vortexed for 15 min at room temperature (RT) and subsequently centrifuged at 21,130× *g* for 10 min. A volume of 500 µL of the supernatant was transferred to a 4.5 mL glass centrifuge tube and evaporated to dryness under a gentle stream of nitrogen, RT (approximately 45 min). The residue was reconstituted in 100 µL acetonitrile/Milli-Q water (50:50, *v*/*v*), transferred to a 96-well plate, and placed in the autosampler for LC-HRMS analysis.

Hippocampal tissue samples (approximately 0.02 g) were homogenized prior to extraction in 100 µL homogenization buffer consisting of 10 mM Trizma (Sigma-Aldrich, T-1503), 320 mM sucrose (Sigma-Aldrich, S0389, Darmstadt, Germany), and 1 mM EDTA (Sigma-Aldrich, ED4SS). Homogenization was performed in 1.5 mL Eppendorf tubes using four stainless steel balls (3 and 5 mm) for 2 cycles of 5 min at 25 Hz in a Retsch Mixer Mill 200 (Retsch, Haan-Gruiten, Germany). Following homogenization, samples underwent an extraction procedure identical to that used for plasma samples. Briefly, samples were acidified with 25 µL of 40% formic acid, followed by protein precipitation with 600 µL of acetonitrile, methanol, and acetic acid (90:10:0.1, *v*/*v*/*v*). Following addition of the precipitation solvent, samples were continuously vortex-mixed for 15 min, RT, and subsequently centrifuged at 21,130× *g* for 10 min. A volume of 550 µL of the resulting supernatant was transferred to a centrifuge tube fitted with a 0.45 µm filter insert and centrifuged at 10,000× *g* for 10 min. Subsequently, 500 µL of the filtered extract was transferred to a 4.5 mL glass centrifuge tube and evaporated to dryness under a gentle stream of nitrogen, RT (approximately 45 min). The residue was reconstituted in 100 µL acetonitrile/Milli-Q water (50:50, *v*/*v*), transferred to a 96-well plate, and placed in the autosampler for LC-HRMS analysis.

### 2.7. Immunohistochemistry

Free-floating brain sections (50 µm) were used for immunohistochemical labeling of Iba-1, NG2, and GST-π. Sections were washed three times for 15 min in TBS (0.05 M, pH = 7.4) and subsequently incubated in TBS, overnight (O/N), with gentle agitation. On the following day, sections were washed three times for 5 min in TBS, followed by three washes for 15 min in TBS containing 0.5% Triton-X-100 (Thermo Scientific, X100-500ML). To reduce nonspecific binding, sections were incubated for 1 h in TBS containing 0.5% Triton X-100 and 10% FBS (Sigma-Aldrich, A38400-01). Sections were then incubated with the following primary antibodies diluted in blocking solution for 48 h at 4 °C: rabbit anti-Iba-1 (Wako, 019-19741, Richmond, VA, USA; 1:600), rabbit anti-NG2 (Sigma Aldrich, ZRB5320; 1:200), or rabbit anti-GST-π (Nordic BioSite, 312, Copenhagen, Denmark; 1:500). Following primary antibody incubation, sections were washed three times in TBS containing 0.5% Triton X-100 and subsequently incubated in hydrogen peroxide/methanol/TBS (1:1:8) to block endogenous peroxidase activity. Sections were washed three times again for 15 min in TBS with 0.5% Triton X-100 and incubated O/N with Envision secondary antibody (anti-rabbit; Aglient, K400311-2, Santa Clara, CA, USA; 1:10). After three additional 15 min washes in TBS containing 1% Triton X-100, immunoreactivity was visualized using diaminobenzidine (DAB; Merck, D5637). Sections were then mounted on gelatine-coated microscope slides. For BrdU-labeling or double-labeling with NG2/BrdU or GST-π/BrdU, sections underwent antigen retrieval in citrate buffer (175.33 g NaCl, 88.23 g NaCitrate in 1L H_2_O, 1:10) for 30 min prior to incubation with rat anti-BrdU (Santa Cruz, Sc-338; 1:100) O/N. After three washes for 15 min in TBS containing 1% Triton-X-100, sections were incubated with biotinylated goat anti-rat secondary antibody (Vector, BA-9400, Newark, CA, USA; 1:200) in TBS containing 20% FBS and 0.5% Triton-X-100 for 1 h. Sections were washed and incubated for 1 h with streptavidin-conjugated alkaline phosphatase (DAKO, P0397, Santa Clara, CA, USA; 1:200) in TBS containing 10% FBS and 0.5% Triton. Following a 15 min rinse in TRIS-HCl buffer, signal was developed in alkaline-phosphatase substrate for 10–30 min [29]. Sections were then washed three times for 15 min in TBS. All sections were counterstained (Iba-1 and double-labeling: Mayer’s hematoxylin, 2 min; GST-π: toluidine blue, 1 min), dehydrated through graded ethanol (70%, 96%, and 99.9%), cleared in xylene, and coverslipped using DPX mounting medium (Sigma-Aldrich, 100579).

### 2.8. Histological Analysis

Whole-slide scans of Iba-1 and GST-π-labeled sections were acquired using a NanoZoomer XR slide scanner (Hamamatsu, Ballerup, Denmark). For GST-π-labeled sections, the hippocampal area was delineated using QuPath (ImageJ plugin v.7) and GST-π^+^ cells were manually counted. The hippocampal formation was defined as the dentate gyrus, cornu ammonis (CA1–CA3) and the subiculum as defined in [30]. The number of Iba-1^+^ microglia was quantified using the automated cell detection algorithm in QuPath. Microglial morphology was analyzed using the AnalyzeSkeleton (2D/3D) Fiji-plugin (v.2.16.0/1.54p). In brief, microglial extensions were marked semi-automatically before images were converted to binary and skeletonized, thereby producing a one-pixel-wide representation of the microglial extensions for branching analysis. Analyses were performed in the hippocampus on three sections per animal and included the following parameters: average cell size, circularity, number of branches, number of junctions, average branch length, and number of triple and quadruple points [31].

Double-labeled NG2/BrdU and GST-π/BrdU cells were quantified using a stereology-based approach using a computer-assisted stereology system (Olympus, Tokyo, Japan) equipped with a 60× objective on an Olympus BH-2 microscope. Cells were counted within a fixed counting frame sampling 41% of the hippocampal area. The total number of double-labeled cells was estimated using the optical fractionator method according to the formula *n* = $\sum Q^{-}\frac{1}{\mathrm{ssf}}\frac{1}{\mathrm{asf}}\frac{1}{\mathrm{tsf}}\;$, where $\sum Q^{-}$ represents the number of counted cells, ssf the section sampling fraction, asf the area sampling fraction, and tsf the thickness sampling fraction [32].

### 2.9. Statistics

Outliers were identified using the Robust regression and Outlier removal (ROUT) outliers test (Q = 1%), and data normality was assessed using the D’Agostino–Pearson test omnibus for sizes ≥8 and the Shapiro–Wilk test for sample sizes *n* < 8. Statistical comparisons between vehicle- and siponimod-treated groups were performed using a two-tailed unpaired Student’s *t*-test for normally distributed data or the Mann–Whitney U-test for non-normally distributed data. For multiple comparisons, a One-Way ANOVA was used for normally distributed data, followed by Dunnett’s multiple-comparisons test or the Kruskal–Wallis test for non-normally distributed data, followed by Dunn’s multiple-comparisons test. Statistical significance was defined as *p* ≤ 0.05. Data are presented as mean ± standard error of the mean (SEM), with individual data points representing individual animals. *p* values are indicated as follows: *p* < 0.05: *, *p* < 0.01: **, and *p* < 0.001: ***. All statistical analyses were performed in GraphPad Prism 10, with graphical assistance from Biorender.com.

### 2.10. Declaration of Generative AI Applied in the Manuscript Preparation Process

This manuscript was composed using the artificial intelligence (AI) tool ChatGPT (OpenAI, v.5.6 Sol). AI was used to refine language and to deliver topic overviews. All AI-assisted contributions have been critically reviewed by the authors, and the authors take responsibility for the content of the manuscript.

## 3. Results

### 3.1. Systemic Siponimod Treatment Induces T Cell Lymphopenia in Mice

**Figure 2:**
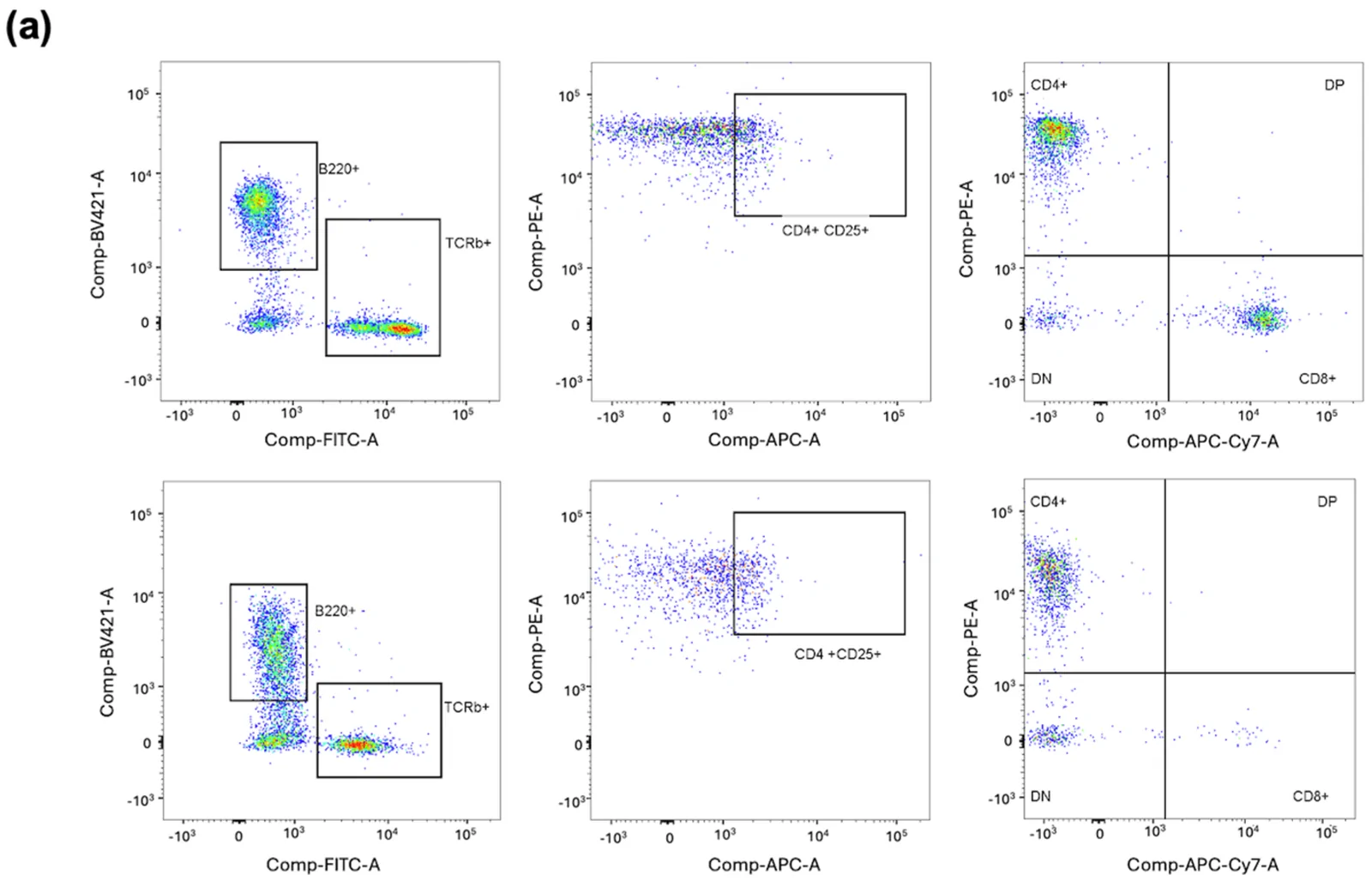

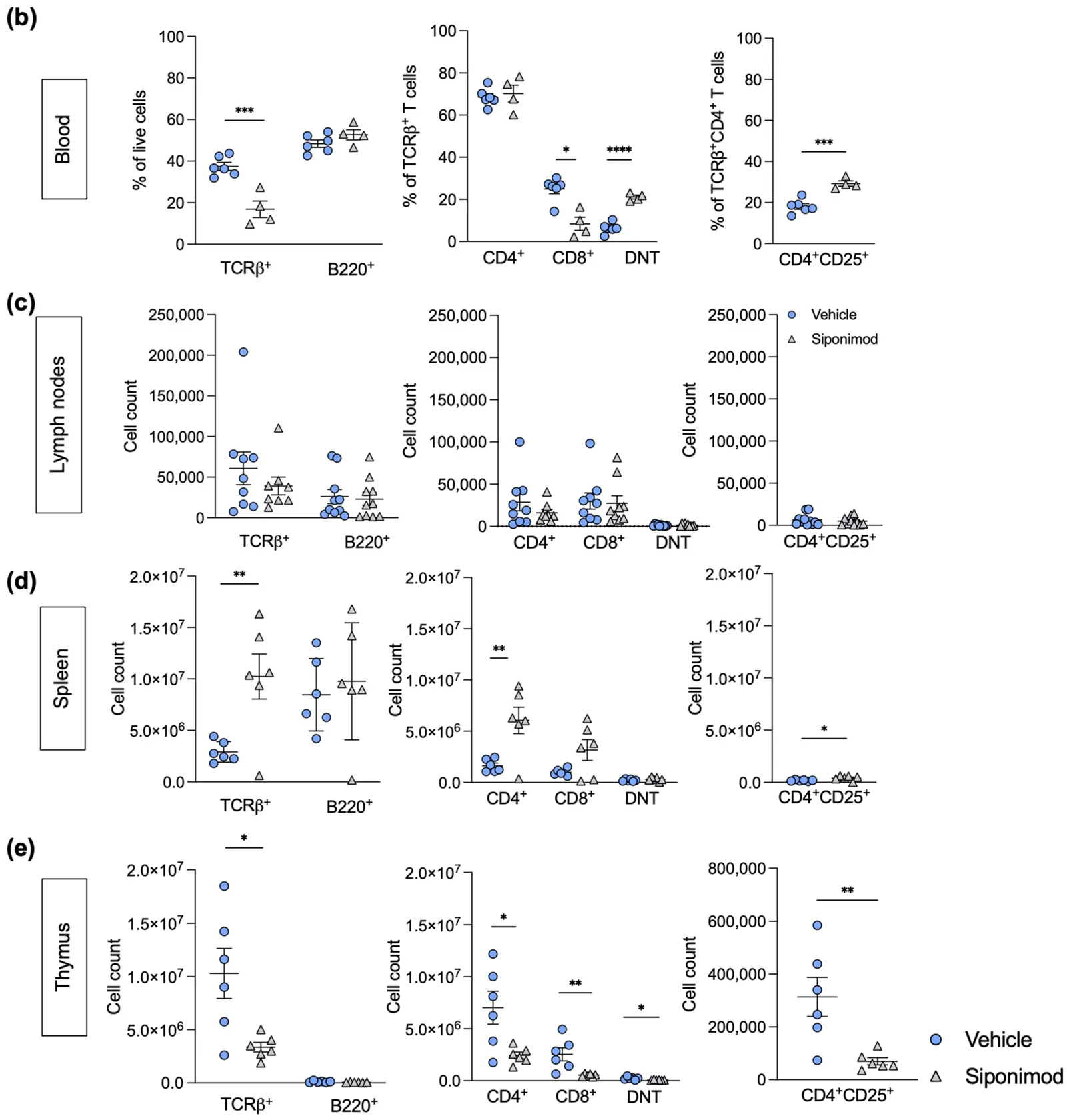
Effect of siponimod on lymphocyte distribution. (**a**) Gating strategy on blood from naïve mice after 19 days treatment with vehicle or siponimod. Only live cells are displayed. (**b**) Flow cytometric analysis of blood-derived T and B cells, CD4^+^ T cells, CD8^+^ T cells, CD4^+^ CD25^+^ T cells, and double-negative T cells (DNT) in vehicle- (*n* = 6) or siponimod (*n* = 4)-treated animals. (**c**–**e**) Flow cytometric analysis of T- and B cells, and T cell subpopulations (CD4^+^, CD8^+^, CD4^+^CD25^+^, and DNT cells) in inguinal lymph nodes (**c**) (vehicle, *n* = 11; siponimod, *n* = 10), spleen (**d**) (vehicle, *n* = 6; siponimod, *n* = 6), and thymus (**e**) (vehicle, *n* = 6; siponimod, *n* = 6) in vehicle- and siponimod-treated mice. Unpaired two-tailed *t*-test or Mann–Whitney U-test, * *p* ≤ 0.05, ** *p* ≤ 0.01, *** *p* ≤ 0.001, **** *p* ≤ 0.0001. Data are shown as mean ± SEM. DNT, double-negative T cells.

Siponimod is known to modulate human immune cell trafficking through S1PR1 signaling [7]. Flow cytometric analysis of blood immune cell populations (Figure 2a,b) revealed that 19 days of treatment with siponimod induced pronounced T cell (TCRβ^+^) lymphopenia in mice, compared to the vehicle-treated controls (37.5% vs. 16.9% of live cells, *p* < 0.001). Within the T cell population, siponimod significantly increased the proportion of double negative T (DNT) cells and CD4^+^CD25^+^ T cells (DNT cells: 6.4% vs. 21.1% of TCRβ^+^ cells, *p* < 0.001; CD4^+^CD25^+^ T cells: 18.1% vs. 29.2% of TCRβ^+^ cells, *p* < 0.001), while cytotoxic CD8^+^ T cells were markedly reduced (26.5% vs. 7.4% of TCRβ^+^ cells, *p* < 0.05). In contrast, neither B cells (B220^+^; 48.4% vs. 52.7% of live cells, ns) nor T helper cell populations (CD4^+^; 68.5% vs. 70.2% of TCRβ^+^ cells, ns) were significantly affected. To assess whether siponimod-induced lymphopenia was associated with lymphocyte retention in lymphoid organs, immune cell populations were additionally analyzed in inguinal lymph nodes (Figure 2c), spleen (Figure 2d), and thymus (Figure 2e). In lymph nodes, no significant differences were observed between treatment groups across any lymphocyte population, including total T cells (60,821 vs. 39,124 cells, ns), B cells (26,123 vs. 23,048 cells, ns), and T cell subpopulations (CD4^+^ T cells: 28,536 vs. 16,247 cells, ns; CD8^+^ T cells: 30,062 vs. 27,410 cells, ns; DNT cells: 1212 vs. 1499 cells, ns; and CD4^+^CD25^+^ T cells: 6317 vs. 4812 cells, ns). In contrast, siponimod treatment resulted in a significant increase in splenic T cells, including CD4^+^ T cells and CD4^+^CD25^+^ T cells (T cells: 2.90 × 10^6^ vs. 10.23 × 10^6^, *p* < 0.01; CD4^+^ T cells: 1.63 × 10^6^ vs. 6.05 × 10^6^, *p* < 0.01; CD4^+^CD25^+^ T cells: 169,754 vs. 415,057, *p* < 0.05). Splenic B cells (8.46 × 10^6^ vs. 9.77 × 10^6^, ns), CD8^+^ T cells (999,238 vs. 3.16 × 10^6^, ns and DNT cells (212,504 vs. 307,956, ns) were not significantly altered by siponimod. In the thymus, siponimod treatment led to a significant reduction in total T cells and all examined T cell subpopulations (T cells: 10.29 × 10^6^ vs. 3.36 × 10^6^, *p* < 0.05; and CD4^+^ T cells: 7.03 × 10^6^ vs. 2.44 × 10^6^, *p* < 0.05; CD8^+^ T cells: 2.54 × 10^6^ vs. 535,828, *p* < 0.01; DNT cells: 235,578 vs. 77,249, *p* < 0.05; CD4^+^CD25^+^ T cells: 313,652 vs. 69,865, *p* < 0.01), whereas thymic B cell numbers were unaffected (115,418 vs. 65,139, ns). Together, these findings demonstrate that oral administration of siponimod at the applied dose effectively induces systemic T cell lymphopenia in mice, consistent with lymphocyte redistribution patterns previously reported in SPMS patients [7].

### 3.2. Siponimod Penetrates the Intact Blood–Brain Barrier

**Figure 3:**
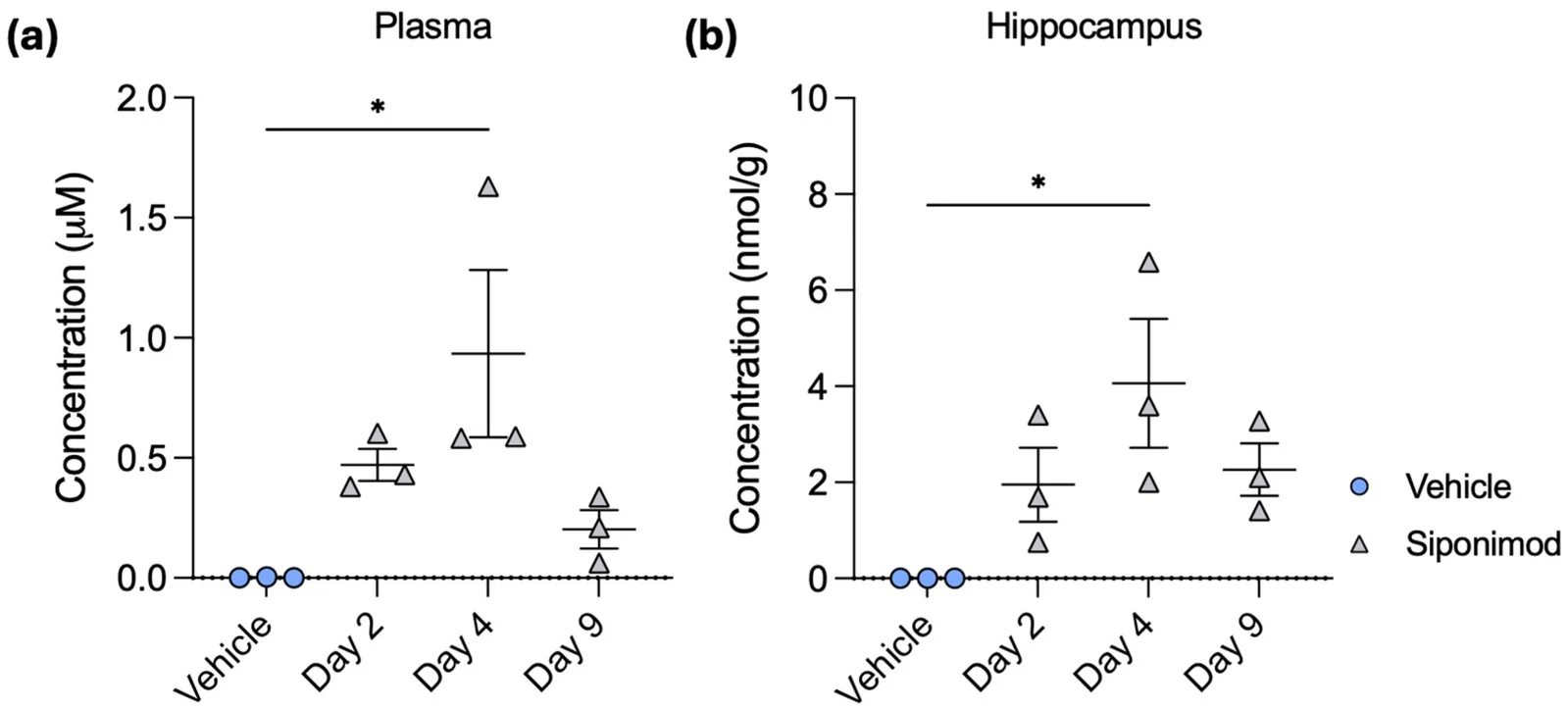
Plasma and brain exposure of siponimod in naïve mice. Siponimod concentrations in plasma (**a**) and hippocampus (**b**) in vehicle- and siponimod-treated animals after 2, 4, and 9 days (*n* = 3 per group). Concentrations were measured approximately 24h after the last administration. Normally distributed data were analyzed using one-way ANOVA followed by Dunnett’s multiple-comparisons test, with each siponimod-treated group compared with the vehicle group. Non-normally distributed data were analyzed using the Kruskal–Wallis test followed by followed by Dunn’s multiple-comparisons test * *p* ≤ 0.05. Data are shown as mean ± SEM.

Siponimod concentrations in plasma samples (Figure 3a) and hippocampal lysates (Figure 3b) were quantified by HRLC-MS following oral administration of siponimod (3 mg/kg) for 2, 4, or 9 consecutive days. Mean plasma concentrations reached 0.5 ± 0.18 μM (*p* < 0.01 compared to vehicle), while hippocampal concentrations reached 2.8 ± 1.63 nmol/g, corresponding to approximately 2.8 ± 1.63 μM (*p* < 0.05 compared to vehicle). The hippocampus-to-plasma concentration ratio was 3.9 ± 1.5 after 2 days, 6.2 ± 4.1 after 4 days, and 16.8 ± 12.2 after 9 days of treatment. These data demonstrate CNS penetration of siponimod and indicate higher hippocampal than plasma concentrations at all treatment durations examined.

### 3.3. Siponimod Does Not Alter Oligodendrocyte Lineage Cells

**Figure 4:**
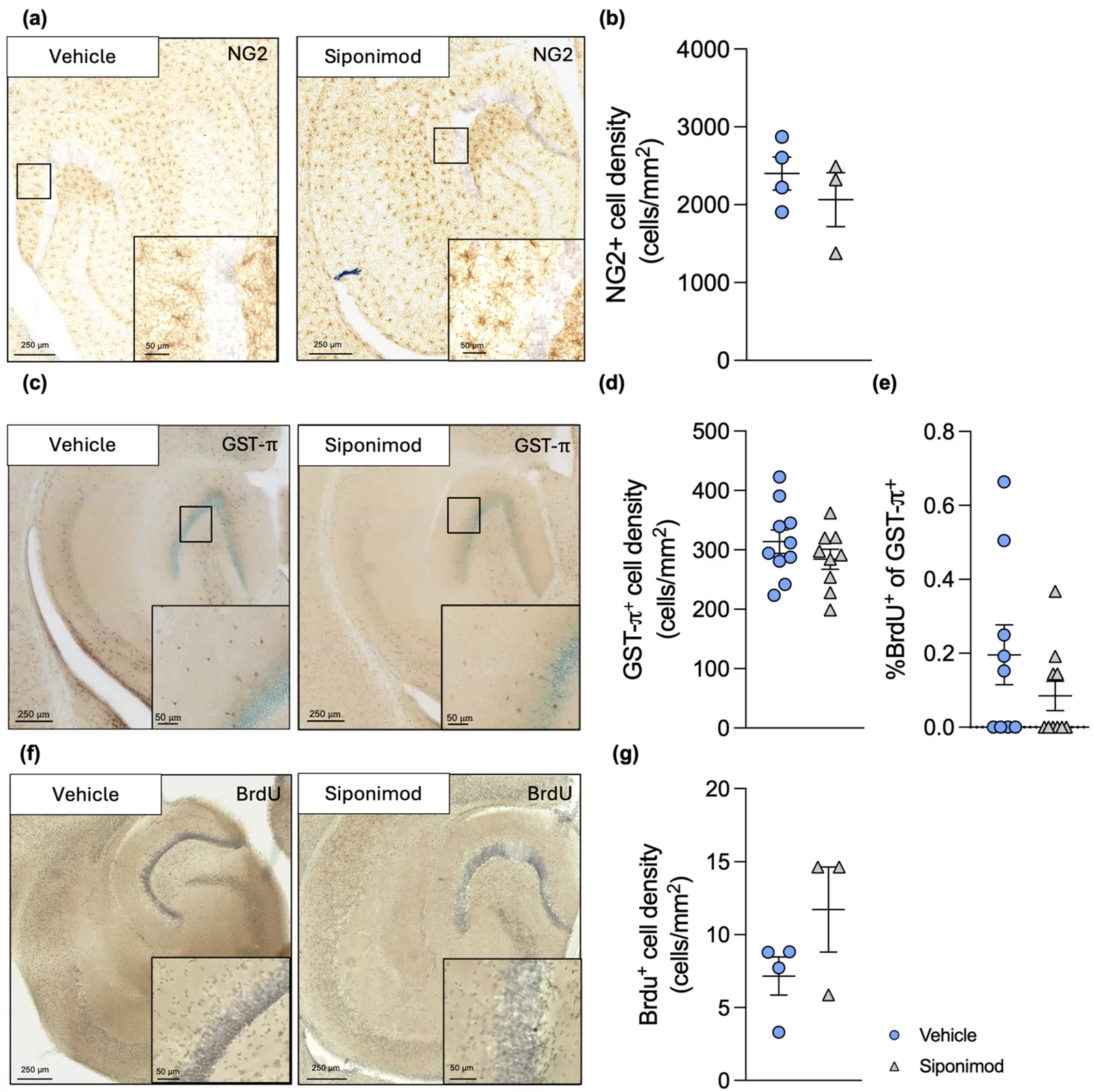
The effect of siponimod on oligodendrocyte-lineage cells in naïve mice. (**a**) Representative images of NG2+ OPCs in the hippocampus of vehicle- and siponimod-treated mice. (**b**) Density of NG2+ cells, counted stereologically in the hippocampus of naïve mice treated with vehicle (*n* = 4) or siponimod (*n* = 3) for 19 consecutive days. (**c**) Representative images of GST-π+ cells in vehicle- and siponimod-treated mouse hippocampi. (**d**) GST-π cell density in the hippocampus of vehicle- (*n* = 10) and siponimod-treated (*n* = 9) mice, manually counted. (**e**) Stereologically counted fraction of proliferating cells (BrdU+) out of the GST- π cell population (vehicle, *n* = 9; siponimod, *n* = 9). (**f**) Representative images of BrdU-labeled cells in mouse hippocampus of vehicle- and siponimod-treated mice. (**g**) BrdU+ cell density of vehicle- (*n* = 4) and siponimod-treated (*n* = 3) mouse hippocampi manually counted. None of the cell density measures were affected by siponimod. Unpaired two-tailed *t*-test or Mann–Whitney U-test. Data are shown as mean ± SEM.

As the myelinating cells of the CNS, the oligodendrocyte lineage cells are key players in the remyelination process [33]. To assess whether siponimod directly affects oligodendrocyte precursor cell (OPC) proliferation and differentiation under physiological conditions, NG2^+^ OPCs and GST-π^+^ mature oligodendrocytes were quantified in the hippocampus in the hippocampal area (Figure 4). Quantitative analysis revealed no significant difference in OPC (NG2^+^) or mature oligodendrocyte (GST-π^+^) densities between vehicle- and siponimod-treated mice (NG2^+^ cells: 2400 vs. 2065 cells/mm^2^, ns; GST-π^+^ cells: 314 vs. 284.2 cells/mm^2^, ns) (Figure 4a–d). To track formation of new oligodendrocyte lineage cells, cells positive for the longitudinal tracer, BrdU, and the percentage of double-labeled NG2^+^/BrdU^+^ and GST-π^+^/BrdU^+^ cells were assessed (Figure 4e–g). Analysis did not detect any NG2^+^/BrdU^+^ cells, indicating that OPC proliferation was negligible under both treatment conditions. While the sampling covered 41% of the hippocampal area, these data suggest that OPC proliferation, if present, represents a very small proportion of dividing cells. Similarly, the percentage of newly formed oligodendrocytes was low and did not differ between groups (0.2% vs. 0.1%, ns). Consistent with this, overall BrdU+ cell density in single-labeled tissue was low and comparable between vehicle- and siponimod-treated mice (7 vs. 12 cells/mm^2^, ns). Collectively, these results indicate that under physiological conditions, siponimod does not alter oligodendrocyte lineage cell density or proliferative activity in the hippocampus.

### 3.4. Siponimod Does Not Affect Microglia Under Physiological Conditions

**Figure 5:**
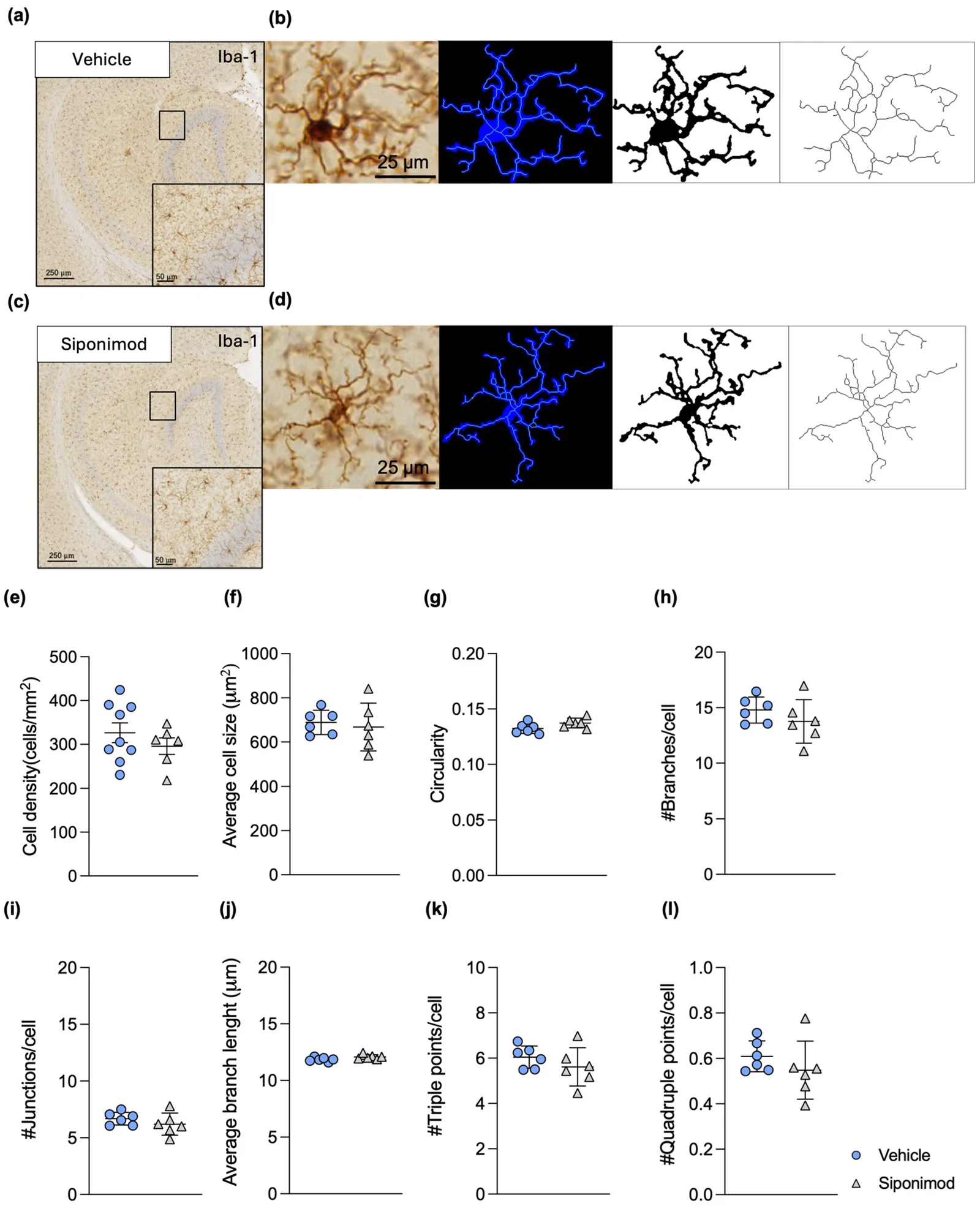
Effects of siponimod on microglia in naïve mice. Representative image of Iba-1-stained microglia in the hippocampus of vehicle-treated (**a**) and siponimod-treated (**c**) mice, with corresponding skeletonized morphology (**b**,**d**). (**e**) Microglial density in the hippocampus (vehicle, *n* = 9, siponimod, *n* = 6) (**f**–**l**) morphological analysis (*n* = 6 per group), including average cell size, circularity, number of branches, number of junctions, average branch length, and number of triple and quadruple points. Unpaired two-tailed *t*-test or Mann–Whitney U-test. Data are shown as mean ± SEM.

To assess whether siponimod affects microglial numbers and morphology under physiological conditions, brain sections from mice treated with vehicle or siponimod for 19 days were immunolabeled with the microglial marker Iba-1, and microglial cell density of the hippocampus was quantified. Quantitative analysis revealed no significant difference in hippocampal microglial density between vehicle- and siponimod-treated mice (327 vs. 296 cells/mm^2^, ns) (Figure 5e). In addition, microglial morphology was analyzed to assess potential treatment-induced changes in reactivity state (Figure 5f–l). Morphological parameters included average cell size, circularity, number of branches, number of junctions, average branch length, and number of triple- and quadruple-branch points. None of the analyzed parameters differed significantly between treatment groups (average cell size: 689 vs. 668 μm^2^, ns; circularity: 0.13 vs. 0.14, ns; number of branches: 14.8 vs. 13.8, ns; number of junctions: 6.7 vs. 6.2, ns; average branch length: 11.9 vs. 12.1 μm, ns; number of triple points: 6.0 vs. 5.6, ns; number of quadruple points: 0.6 vs. 0.5, ns). Together, these findings indicate that siponimod does not alter microglial density or morphology in the hippocampus of mice.

### 3.5. Siponimod Does Not Affect Locomotor Activity or Spatial Memory

**Figure 6:**
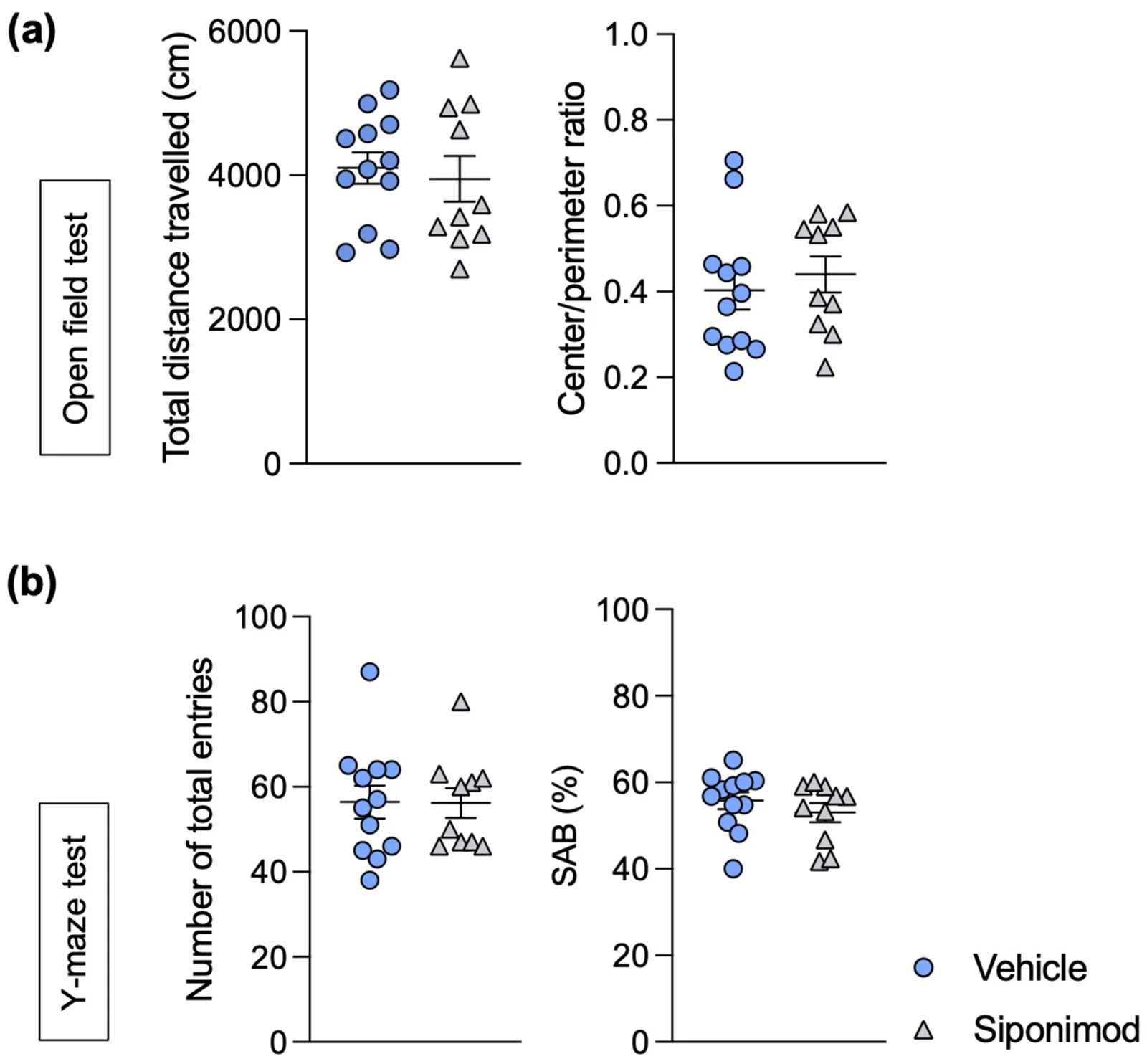
The effect of siponimod on behavior in naïve mice. Y-maze (**a**) and open field (**b**) behavioral tests were performed after 19 days of treatment with siponimod (*n* = 10) or vehicle (*n* = 12). Unpaired two-tailed *t*-test. Data are shown as mean ± SEM. SAB, spontaneous alternation behavior.

Given that siponimod has been reported to improve cognitive performance and working memory in patients with SPMS [34] and that neuroinflammation of the hippocampus has been reported in MS [35], we assessed locomotor activity and spatial working memory in mice using the open field and Y-maze tests (Figure 6). The open field test was used to evaluate locomotor activity and anxiety-related behavior, measured as total distance travelled and the center-to-perimeter ratio (Figure 6a). Spatial working memory was assessed using the Y-maze test, with outcomes including total number of arm entries and %SAB (Figure 6b). None of the behavioral parameters differed between vehicle- and siponimod-treated mice. Total distance travelled in the open field was comparable between groups (4100 vs. 3946 cm, ns), as was the center-to-perimeter ratio (0.4 vs. 0.4, ns). Similarly, siponimod treatment did not affect exploratory activity or spatial working memory in the Y-maze, as reflected by identical numbers of arm entries (56 vs. 56, ns) and comparable levels of SAB (56% vs. 53%, ns). The results indicate that siponimod does not alter locomotor activity, anxiety-related behavior, or spatial working memory in mice.

## 4. Discussion

In the present study, we investigated the systemic and CNS-specific effects of siponimod in naïve mice treated orally with 3 mg/kg siponimod for 19 consecutive days. Siponimod is a selective S1PR1 and -5 modulator that primarily targets immune cell trafficking but has also been proposed to exert direct effects on glial cells within the CNS. By combining pharmacokinetic analyses, flow cytometry, immunohistochemistry, and behavioral testing, we aimed to distinguish peripheral immunomodulatory actions from potential direct CNS effects under physiological conditions. This is, to our knowledge, the first demonstration of pronounced siponimod-induced alterations in peripheral T cell distribution and systemic lymphopenia under physiological conditions, while no measurable effects were observed on oligodendrocyte lineage cells, microglia, or behavior in naïve mice. Consistent with the established mechanism of action of S1PR modulators, siponimod treatment resulted in pronounced T cell lymphopenia in blood. Binding of siponimod to S1PR1 on lymphocytes induces receptor internalization and degradation, thereby blocking lymphocyte egress from lymphoid organs and reducing circulating lymphocyte numbers [7,25]. Similar reductions in circulating lymphocytes have been reported in patients with SPMS treated with siponimod [7,8,36]. In agreement with these findings, our flow cytometric analyses demonstrated a marked reduction in circulating T cells after 19 days of treatment. Interestingly, this depletion preferentially affected CD8^+^ T cells, whereas CD4^+^ T cells and B cells remained largely unaffected. In contrast, clinical studies in SPMS patients treated with siponimod have reported broader lymphopenia affecting both CD4^+^ and CD8^+^ T cells, as well as B cells [7]. These differences may reflect MS- or species-specific differences in lymphocyte trafficking or the dependency of lymphocyte subsets on S1PR1 signaling. Experimental studies have suggested that lymphocyte subsets differ in their reliance on S1PR1-mediated migration, as CD8^+^ T cells appear to depend more strongly on S1PR1 signaling for egress from secondary lymphoid organs, whereas B cells rely on additional mechanisms for circulation [5,37]. Supporting this notion, in vitro migration assays have demonstrated that CD8^+^ T cells respond more robustly to S1P gradients than CD4^+^ T cells [5,37]. In addition to the preferential depletion of CD8^+^ T cells, we observed an increase in DNT cells and CD4^+^CD25^+^ T cells in circulation. Regulatory T cells have previously been reported to increase in the blood of siponimod-treated SPMS patients [7,8], suggesting that the CD4^+^CD25^+^ population detected in the present study may represent an expansion of regulatory T cells. The marked increase in DNT cells may reflect a compensatory response to T cell depletion following siponimod treatment, although the functional significance of this population remains unclear.

Siponimod-induced lymphopenia is widely believed to result from the retention of lymphocytes within lymphoid organs following inhibition of S1PR1-dependent egress [7,25]. However, the specific lymphoid compartments responsible for this redistribution remain incompletely understood. Previous studies have reported decreased lymphocyte numbers in lymph nodes, spleen, and thymus following siponimod administration [24,38], suggesting that lymphocyte dynamics may differ between anatomical compartments. To further investigate this, we analyzed lymphocyte populations in lymph nodes, spleen, and thymus of siponimod- and vehicle-treated mice. Interestingly, we observed no enrichment of lymphocyte populations in lymph nodes, while all T cell subpopulations were significantly reduced in the thymus. In contrast, splenic T cell populations, including total T cells, CD4^+^ T cells, and CD4^+^CD25^+^ T cells, were significantly increased following siponimod treatment. These findings differ from previous studies describing reduced splenic lymphocyte numbers after siponimod treatment [24,38,39]. Such discrepancies may reflect differences in experimental design, treatment duration, or disease context. Our data therefore suggest that the spleen may represent an important compartment for lymphocyte redistribution in mice under siponimod treatment.

For siponimod to exert direct effects within the CNS, the drug must cross the BBB. Pharmacokinetic analyses in this study demonstrated that siponimod readily penetrates the BBB and accumulates within the hippocampus at concentrations exceeding those measured in plasma. These findings are consistent with previous in vivo studies reporting higher CNS concentrations of siponimod compared with plasma levels [13,40]. Siponimod’s lipophilic nature and relatively low molecular weight facilitate its CNS penetration [41]. Although the plasma concentrations observed in the present study exceeded those reported in serum from healthy human subjects treated with siponimod (30.4 ng/mL following continuous oral administration of 2 mg siponimod once daily) [42], the results demonstrates that the chosen dosing regimen achieved systemic exposure sufficient for CNS penetration. Given the robust CNS exposure observed, we next investigated whether siponimod directly affects glial cell populations under physiological conditions. Previous studies have suggested that siponimod exerts neuroprotective and pro-regenerative effects within the CNS, including enhanced remyelination, increased oligodendrocyte survival, and modulation of microglial reactivity [12,13,17,43,44]. In experimental models of demyelination, siponimod has been shown to promote remyelination and increase the expression of myelin components through S1PR1-mediated effects on microglia and astrocytes or S1PR5-mediated effects on oligodendrocyte lineage cells [11,13,15,16,45]. However, most of these studies were conducted in inflammatory or demyelinating disease models. In contrast, our analyses in mice revealed no detectable effects of siponimod on oligodendrocyte lineage cells. Neither OPC density nor mature oligodendrocyte density differed between treatment groups, and proliferative activity within the oligodendrocyte lineage remained extremely low under both conditions. Similarly, siponimod treatment did not alter microglial density or morphology in the hippocampus.

Morphological analysis using skeletonization parameters, including branch number, branch length, junctions, and circularity, revealed no significant differences between treatment groups. These findings suggest that, in the absence of pathology, siponimod does not markedly influence glial cell populations or activation states. The neuroprotective and remyelinating effects reported in disease models may therefore depend on inflammatory cues or immune–glia interactions rather than direct activation of glial S1PR signaling under physiological conditions. In line with this, S1PRs are differentially expressed in MS lesions [46]. Findings from the present study suggest that the clinical benefits of siponimod are likely mediated indirectly through modulation of peripheral immune responses rather than direct CNS effects.

To assess whether siponimod affected behavioral outcomes in mice, locomotor activity and spatial working memory were evaluated using the open field and Y-maze tests. Neither locomotor activity nor anxiety-related behavior differed between siponimod- and vehicle-treated animals in the open field test. Similarly, siponimod treatment did not affect exploratory activity or spatial working memory in the Y-maze test. These findings indicate that siponimod does not affect general behavior or cognitive performance in mice and provide important information on the safety of the drug. In contrast, improved cognitive performance has been reported in SPMS patients treated with siponimod [34]. This was, however, shown in a subset of SPMS patients characterized by either clinical or radiological disease activity, indicating ongoing inflammation of the CNS [47,48]. Such improvements likely reflect disease-specific effects related to reduced neuroinflammation or restoration of neural network function rather than direct pharmacological effects within the CNS [47,48].

This study is not without limitations. While immunohistochemistry combined with manual quantification provides high spatial resolution and enables detailed morphological assessment of glial cells in situ, it is inherently limited by sampling constraints, potential observer bias, and reduced sensitivity to subtle changes. In addition, a substantial number of animals were excluded from the NG2 analysis because of insufficient tissue quality, and three animals were excluded from the morphological analysis, thereby reducing the effective sample size and statistical power. This is particularly relevant for the NG2 analysis and increases the possibility that small treatment-related differences remained undetected. Accordingly, the absence of significant differences should be interpreted as a lack of detectable effects under the present experimental conditions rather than definitive evidence of no biological effect. Furthermore, the present analyses primarily assessed cell density, proliferation, and morphology and therefore do not exclude more subtle molecular or functional effects of siponimod on microglia or oligodendrocyte lineage cells. Changes in cellular phenotype may occur without detectable alterations in cell number or gross morphology. Additional analyses of molecular markers related to microglial activation and homeostasis, oligodendrocyte differentiation, and myelin integrity would therefore be required to determine whether siponimod induces more subtle changes in these cell populations under physiological conditions. A further limitation of the present study is the exclusive use of female mice. Females were selected because MS has a marked female predominance [23]; however, biological sex is known to influence immune responses and disease expression in MS and experimental MS [23,49,50,51]. Women are more frequently affected by MS, whereas men have in several studies been associated with faster disability progression and greater neurodegenerative burden [23,49]. Sex-dependent differences in immune responses have also been reported in experimental autoimmune encephalomyelitis [50,51]. Accordingly, the systemic and CNS effects of siponimod observed here may not be directly generalizable to male mice, and future studies including both sexes are needed to determine whether treatment responses differ by sex.

## 5. Conclusions

In summary, oral administration of siponimod at 3 mg/kg for 19 days induced pronounced peripheral T cell lymphopenia and altered lymphocyte distribution across lymphoid organs in naïve mice. Although siponimod readily penetrated the CNS and achieved hippocampal concentrations consistently exceeding those in plasma, no measurable effects were observed on oligodendrocyte lineage cells, microglia, or behavior. These findings indicate that under physiological conditions, the primary actions of siponimod are restricted to immunomodulation in mice. The neuroprotective and remyelinating effects reported in disease models and clinical settings therefore likely depend on interactions with inflammatory processes or immune–glia signaling pathways, rather than direct activation of glial receptors in the healthy brain. Understanding this could pave the way for optimizing treatment strategies, better interpretation of clinical outcomes, and guiding development of next-generation S1PR modulators.

## Data Availability

The datasets included in the current study are available from the corresponding author upon reasonable request, as they have not been deposited in a public repository.

## Abbreviations

The following abbreviations are used in this manuscript:

ANOVA	Analysis of variance
APC	Allophycocyanin (fluorochrome)
APC-Cy7	Allophycocyanin-Cyanine 7 (fluorochrome)
asf	Area sampling fraction
BBB	Blood–brain barrier
BrdU	Bromodeoxyuridine
CA	Cornu ammonis
CD	Cluster of Differentiation
CNS	Central nervous system
D-PBS	Dulbecco’s PBS
DAB	Diaminobenzidine
DNT	Double-negative T cells
FACS	Fluorescence-activated cell sorting
FBS	Fetal bovine serum
FITC	Fluorescein isothiocyanate (fluorochrome)
flow cyt.	Flow cytometry
GST	Glutathione S-transferase
HBSS	Hank’s balanced salt solution
Iba1	Ionized calcium-binding adapter molecule 1
IHC	Immunohistochemistry
i.p.	Intraperitoneal
LC-HRMS	Liquid chromatography and high-resolution mass spectrometry
LC-MS	Liquid-chromatography mass spectroscopy
*m*/*z*	Mass-to-charge notation
MS	Multiple sclerosis
Na-CMC	Sodium carboxymethyl cellulose
NG2	Neural/glial antigen 2
ns	Not significant
O/N	Overnight
OPC	Oligodendrocyte precursor cell
PBS	Phosphate-buffered saline
PE	Phycoerythrin (fluorochrome)
PerCP-Cy5.5	PerCP-Cyanine5.5 (fluorochrome)
PFA	Paraformaldehyde
PVP	Polyvinylpyrrolidone
RT	Room temperature
S1P	Sphingosine-1-phosphate
S1PR	Sphingosine-1-phosphate receptor
SAB	Spontaneous alternation behavior
SEM	Standard error of the mean
Sip	Siponimod
SPMS	Secondary progressive multiple sclerosis
ssf	Section sampling fraction
TBS	Tris-buffered saline
TCR	T cell receptor
tsf	Thickness sampling fraction
UHPLC	Ultra high-pressure liquid chromatography

## Appendix A

**Table A1:** Antibodies for lymphocyte panel.

Antibody	Fluorochrome	Host (Clone)	Dilution	Source, Cat. No.
CD45	PerCP-Cy^TM^5.5	Rat (30-F11)	1:100	Biolegend, 103132
TCR*β*	FITC	Rat (H57-597)	1:200	Biolegend, 109206
CD4	PE	Rat (RM4-5)	1:300	Biolegend, 100512
CD8a	APC-Cy7	Rat (53-6.7)	1:200	Biolegend, 100714
CD25	APC	Rat (PC61)	1:300	Biolegend, 102012
CD45R/B220	Pacific Blue^TM^	Rat (RA3-6B2)	1:200	Biolegend, 103227
